# Evaluation of Remote Delivery of Passive Integrated Transponder (PIT) Technology to Mark Large Mammals

**DOI:** 10.1371/journal.pone.0044838

**Published:** 2012-09-11

**Authors:** W. David Walter, Charles W. Anderson, Kurt C. VerCauteren

**Affiliations:** United States Department of Agriculture, Animal and Plant Health Inspection Services, Wildlife Services, National Wildlife Research Center, Fort Collins, Colorado, United States of America; Texas A&M University-Corpus Christi, United States of America

## Abstract

Methods to individually mark and identify free-ranging wildlife without trapping and handling would be useful for a variety of research and management purposes. The use of Passive Integrated Transponder technology could be an efficient method for collecting data for mark-recapture analysis and other strategies for assessing characteristics about populations of various wildlife species. Passive Integrated Transponder tags (PIT) have unique numbered frequencies and have been used to successfully mark and identify mammals. We tested for successful injection of PIT and subsequent functioning of PIT into gelatin blocks using 4 variations of a prototype dart. We then selected the prototype dart that resulted in the least depth of penetration in the gelatin block to assess the ability of PIT to be successfully implanted into muscle tissue of white-tailed deer (*Odocoileus virginianus*) post-mortem and long-term in live, captive Rocky Mountain elk (*Cervus elaphus*). The prototype dart with a 12.7 mm (0.5 inch) needle length and no powder charge resulted in the shallowest mean (± SD) penetration depth into gelatin blocks of 27.0 mm (±5.6 mm) with 2.0 psi setting on the Dan-Inject CO_2_-pressured rifle. Eighty percent of PIT were successfully injected in the muscle mass of white-tailed deer post-mortem with a mean (± SD) penetration depth of 22.2 mm (±3.8 mm; n = 6). We injected PIT successfully into 13 live, captive elk by remote delivery at about 20 m that remained functional for 7 months. We successfully demonstrated that PIT could be remotely delivered in darts into muscle mass of large mammals and remain functional for >6 months. Although further research is warranted to fully develop the technique, remote delivery of PIT technology to large mammals is possible using prototype implant darts.

## Introduction

Researchers are often challenged with identifying methods for humane and efficient marking and identification of free-ranging wildlife for a variety of research and management purposes. The use of Passive Integrated Transponder technology could be a more efficient method for mark-recapture analysis of various wildlife species. Passive Integrated Transponder tags (PIT) have unique numbered frequencies and have been used to successfully mark and subsequently identify mountain hare (*Lepus timidus*), desert tortoise (*Gopherus agassizii*), Wryneck (*Jynx torquilla*), and Adélie penguins (*Pygoscelis adeliae*) at PIT readers comparable to a recapture [1]–[4]. Although the distance at which readers can record PIT are currently limited, PIT have been successfully used to detect animals at distances ranging from 3 cm to 1 m [1], [3], [5]. Readers of PIT have been set up along culverts, feeding stations, nest boxes, and water sources to document presence of or use by animals marked with PIT and portable energy sources are available to provide power to readers [1], [2], [4], [6]. Use of PIT offers numerous advantages to other conventional methods such as a lower expense for capture and marking large numbers of individuals compared with radiocollars or external tags, inexpensive units ($8 per PIT), safer for wildlife and researchers/biologists than capture, and likely are aesthetically more appealing to viewers of wildlife compared to radiocollars.

Most mark-recapture studies are limited by the number of animals monitored, which is limited by the expense of capture for marking, radiotelemetry equipment, and monitoring protocols and methods (e.g., helicopter counts, cameras; [7]–[9]). To date, all use of PIT has required capture and injection of PIT by hand [2], [10]. Remote delivery of PIT via CO_2_-pressured dart rifle to mark individual large mammals has not been evaluated. Research on use of PIT to mark mammals would benefit many agencies searching for cost-effective methods for mark/recapture studies to monitor populations of large mammals. For example, in game mammals like elk (*Cervus elaphus*), deer (*Odocoileus spp*.), and black bear (*Ursus virginianus*), remote delivery of PIT could be followed by scanning of injection site at check stations after being harvested by hunters. Marking remotely with PIT would be a random sample of the population, similar to capturing or trapping large mammals for marking with ear-tags or radiocollars provided that sampling designs were set-up and followed. More importantly, remote delivery of PIT would be less invasive to study animals, safer for researchers, and potentially more practical and economical. Use of PIT may provide more detail on harvest rates, population estimates, and movements not previously possible due to the expense and logistics of radiotelemetry technology and monitoring of individuals.

As with any invasive technology that can be used to permanently mark large mammals, humane and behavioral concerns for the study animal should be considered [11]. Intra-muscular injection of PIT in Loggerhead (*Caretta caretta*) and Kemp's ridley (*Lepidochelys kempii*) seaturtles and American eels (*Anguilla rostrata*) was considered superior to subcutaneous injection because quick encapsulation rendered PIT more stable with less migration than in subcutaneous injections [12], [13]. Subcutaneous injection of PIT in small- and medium-sized mammals resulted in minimal or no infections at the injection site and minimal migration in the body away from the initial injection site [10], [14], [15]. Implanting PIT into various tissues other than subcutaneously also resulted in minimal to no infections in hooved mammals [16].

Remote delivery of PIT would likely be comparable to remote darting and injection of chemical immobilization drugs that is routine in wildlife research and no detrimental effects from infections caused by remote darting have been documented [17]–[19]. Vaccines delivered in biobullets have been injected and monitored with minimal injection-site trauma, abscesses, or tissue damage detected in free-ranging wildlife or domestic cattle [20], [21]. Skin biopsy darts for DNA sampling that remove a 3 mm×10 mm deep section of skin and tissue in a tearing manner resulted in minimal bleeding, no behavioral effects, and has been tested on pronghorn (*Antilocapra americana*), African elephants (*Loxodonta africana*), and a variety of primates [22]–[24]. Remote darting for chemical immobilization, biobullet delivery, or biopsies has been approved by institutional care and use committees and any resulting trauma would be much less stressful or damaging to study animals than capture, manual restraint, or anesthesia [25].

This was a preliminary study with the overall goal of evaluating whether PIT tags can be delivered remotely. Our study was designed in phases to address animal welfare and to prevent use of technology on a live animal should it fail in controlled tests. To this end, we had 3 primary objectives: 1) to test prototype darts, 2) inject PIT into muscle tissue, and 3) to assess longevity of PIT functionality in a mammal. Specifically, our first objective was to select the prototype dart design that would inject PIT upon impact the shallowest depth to determine if the idea was feasible prior to animal testing. To test that darts were able to inject PIT into muscle tissue as it did in gelatin, our second objective was to use the selected prototype dart from objective 1 to assess the ability of PIT to be successfully implanted into muscle tissue using remote delivery into hind quarters of white-tailed deer post-mortem. Our third objective was to determine the potential for use of PIT in free-ranging wildlife by assessing remote delivery and longevity of functioning PIT in live, captive elk.

## Materials and Methods

### Prototype dart trials

**Figure 1:**
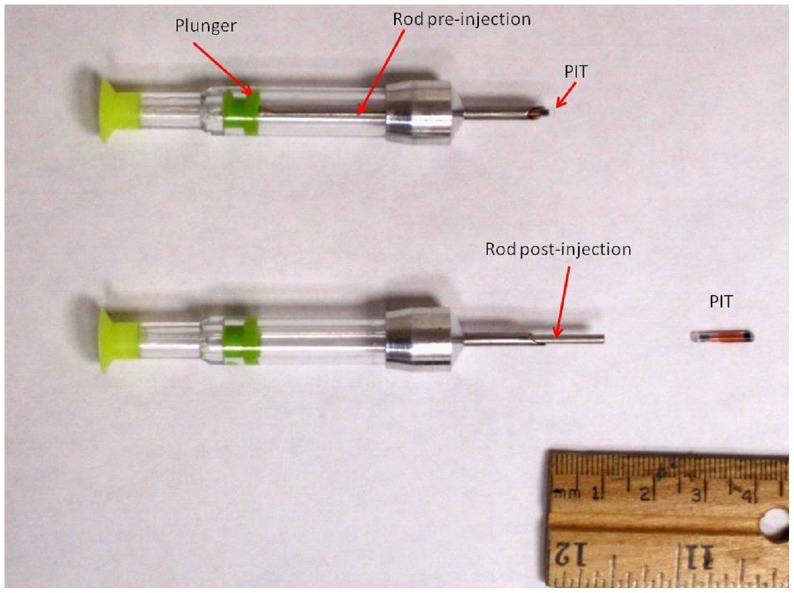
Prototype darts were used to assess remote delivery of Passive Integrated Transponder tags (PIT) into a gelatin block. Prototype darts included a steel rod used to expel the PIT with the aid of a rubber plunger. Darts with and without powder charges behind the rubber plunger were used to determine the influence of injection speed on penetration depth of PIT into tissue.

We prepared a mixture of 5% gelatin powder and 1200 ml of water on a hot plate heated to 230°C and stirred with a metal spatula until the powder was completely dissolved in solution. We poured the mixture into 2-ply plastic bags confined by a box to create a square mold with dimensions of 15×10×15 cm. We refrigerated the entire mold to allow gel solution to solidify prior to removing bag from the mold (hereafter referred to as gelatin block). We set up a wooden case to contain the gelatin block equipped with a digital video camera to record impact of dart and injection of PIT into the gelatin block. We used 2.1 mm×12.5 mm glass PIT read at 134.2 kHz (ISO FDX-B; Biomark®, Boise, Idaho). We used a Dan-Inject rifle model JM Standard (rifle; Dan-Inject of North America, Fort Collins, CO, USA) to project the loaded prototype implant darts (Pneu-Dart, Inc., Williamsport, PA, USA) and to inject PIT into the gelatin block upon impact. The prototype implant dart was 1 cc (1 milliliter equivalent) with a 12-gauge needle and contained a rod within the needle to assist in injection of PIT into the gelatin block (Fig. 1). Gelatin blocks were inspected for success of delivery and PIT functioning was assessed by reading with a Pocket Reader EX with a reading range of 5.1–11.4 centimeters or a FS2001F-ISO with a reading range of 22.9–36.8 centimeters (reader; Biomark®, Boise, Idaho; hereafter both referred to as a reader).

To maximize the efficiency of the reader to detect PIT, we wanted to implant PIT as shallow in the muscle as possible because remote delivery of PIT subcutaneously would not be possible. To address this issue, we used 10 replicates of 4 prototype darts and measured the depth PIT penetrated the gelatin block fired using a 2.0 pounds/inch^2^ (psi) setting. We did not vary psi throughout the trials for consistency of each dart type and because number of darts available limited the number of trials for each objective. The 4 prototype darts were:

1. 1 cc, 12 gauge needle, 25.4 mm (1 inch) needle length with powder charge (Dart 1).
2. 1 cc, 12 gauge needle, 25.4 mm (1 inch) needle length without powder charge (Dart 2).
3. 1 cc, 12 gauge needle, 12.7 mm (0.5 inch) needle length with powder charge (Dart 3).
4. 1 cc, 12 gauge needle, 12.7 mm (0.5 inch) needle length without powder charge (Dart 4).

**Figure 2:**
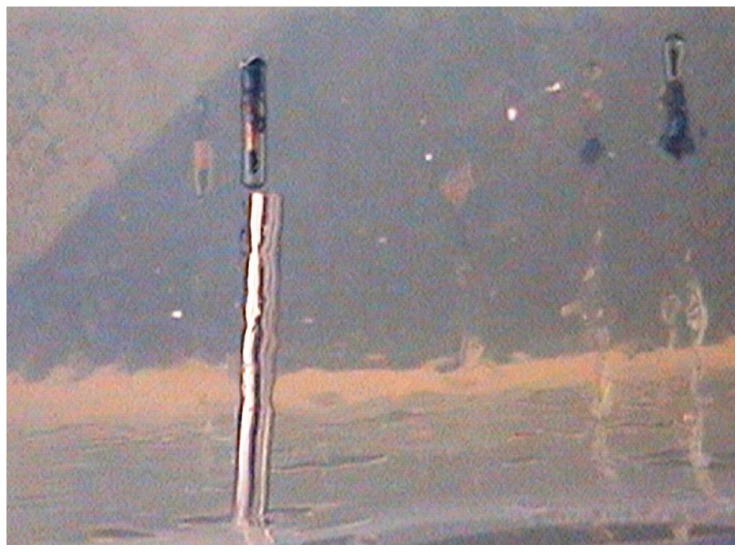
Penetration of Passive Integrated Transponder tags (PIT) into a gelatin block after being injected from a prototype dart. A probe was inserted into the path the PIT followed and length was measured to determine the depth that PIT penetrated the gelatin block. The prototype dart with the shortest depth of penetration was considered most suitable for remote darting into tissue.

We selected darts similar to a study on wound characteristics from remote darting of immobilization drugs that showed longer needle length and rapid injection (i.e., with powder charges) resulted in more contamination of wound cavities compared to shorter needle length and slow injection [26]. After remote delivery into gelatin blocks, we inserted a probe into the path of the dart until it reached the base of the PIT then measured the depth to the nearest millimeter (Fig. 2). We conducted a one-way analysis of variance on depth of injection and the prototype dart that penetrated the shallowest depth into gelatin blocks was used for remote delivery in the remainder of our objectives. We set significance of our statistical test at *P*≤0.05.

### Remote delivery into tissue

To determine depth and ability of PIT to implant in animal tissue, we darted hind quarters of 4 white-tailed deer that were collected post-mortem (cadavers) with 5 PIT per hind quarter at 20 meters. We used darting of cadavers instead of gelatin blocks covered in hide because covering with a hide is difficult to mimic the taunt nature of hide and muscle and could result in only partial penetration of the dart body through hide [26]. White-tailed deer were euthanized as part of a collaborative study separate from this research making them available for our use within 2 hours post-euthanization (Colorado State University Animal Care and Use Research/Teaching Protocol 09128A01). We loaded the PIT into the prototype implant dart that injected the PIT the shallowest depth in the gelatin block and remotely delivered PIT from the rifle. After testing a few shots on cadavers at 2.0 psi, we increased psi to 2.5 to ensure complete injection of dart needle tip into muscle and through skin and subcutaneous adipose tissue. Velocity of dart influences needle penetration but not depth of PIT injection past the tip of the dart needle. After remote delivery of PIT, cadavers were scanned with the reader to determine successful delivery and functionality by reading PIT. We then skinned the cadaver to reveal the entry of PIT into muscle and measure distance PIT penetrated muscle using a probe and ruler to the nearest millimeter. Although we marked entry of PITs, following the path through muscle tissue for accurate depth measurement was not possible for a majority of trials. Muscle tissue was not clear in nature like the gelatin and the microscopic path PIT followed upon penetration, similar to hand injection in seaturtles [13], proved difficult to follow post-darting. Therefore, an accurate depth could not be measured because too much tissue was needed to be removed to locate the PIT. We attempted in using a PIT reader to aid us in finding these tags within the muscle tissue but the search pattern was not narrow enough to negate excess tissue removal. Our primary goal for all PIT injected, however, was to find the PIT in tissue for further inspection to determine condition and functioning of PIT post-injection. All PIT were recovered in cadavers to determine PIT functionality before and after excision out of muscle.

### Longevity in a captive large mammal

We remotely darted 14 captive elk, individually identified with numbered eartags, in the hind quarter using the rifle at about 20 meters at 2.5 psi with the prototype implant dart used in muscle mass of deer cadavers. The captive elk are maintained under USDA standard operating procedures for captive wildlife (National Wildlife Research Center Animal Care and Use Committee Quality Assurance protocol 1487). The captive elk were all males that were 3 years-of-age during the study and were free-ranging behind a 2.0-m tall fence covering 7 ha. The dart needle was sealed with wide-spectrum antibiotic ointment prior to injection to help prevent infection post-darting and to hold PIT inside dart needle during remote delivery. Sealing the dart needle with antibiotic ointment was necessary because PIT occasionally fell out of the dart during flight in preliminary evaluations of remote delivery. The 3 failed first attempts had the dart eject from the elk hind quarter prior to successfully implanting the PIT while darts from the 11 successful deliveries remained in the elk for >5 minutes; we darted elk with PIT a second time for the 3 failed attempts. Twenty-four hours after remote injection, elk were run through a squeeze chute handling system and the reader was scanned over the injection site to determine successful injection and functioning of PIT. The squeeze chute was only used during handling of captive elk and was not accessible to captive elk or free-ranging wildlife when not in use for this research. Data on injection site condition (e.g., presence/absence of abscess), location of PIT, and frequency of PIT was recorded for each elk. If we were unable to read the PIT, the elk was remotely darted with an additional PIT with subsequent reading of PIT about 30 days later. We visually monitored elk within 20 m daily for 21 days to determine if any change in gate or condition of elk occurred in response to darting with PIT. We then determined proper PIT functioning in elk monthly by a combination of squeeze chutes or scanning with reader by hand during feeding operations. Use of all elk for the purposes of our study was approved by the National Wildlife Research Center Animal Care and Use Committee Quality Assurance protocol 1802.

## Results

### Prototype dart trials

**Figure 3:**
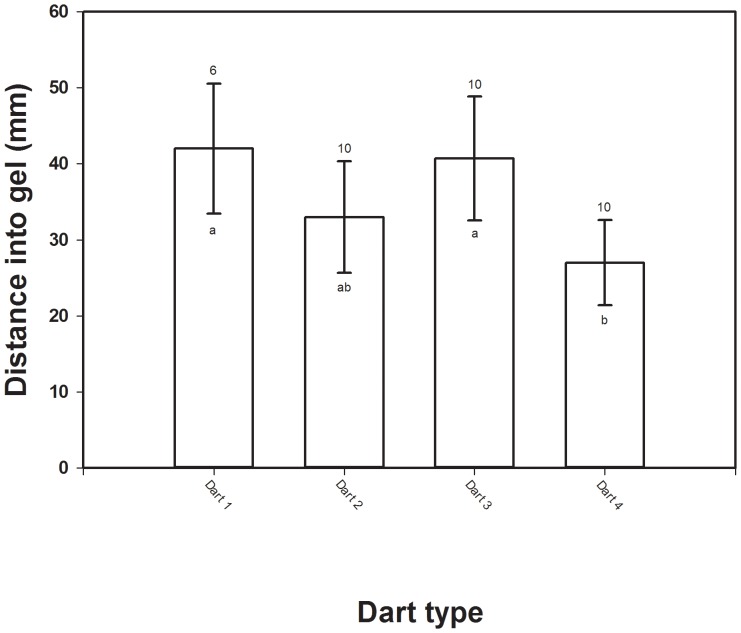
A comparison of depth of penetration for 4 prototype darts used to remotely inject Passive Integrated Transponder tags (PIT) into a gelatin block. Mean (± SD bars) depth PIT penetrated the gelatin block by the 4 prototype darts. Number above bars is sample size and similar letters below bars indicated no difference in depth PIT penetrated the gelatin block at *P*<0.05.

Darts 1 and 3 that included a powder charge penetrated the gel block the deepest with mean (± SD) penetration depths of 40.7 mm (±8.1 mm) and 42.0 mm (±8.5 mm), respectively (Figure 3). Dart 2 with no powder charge had a mean (± SD) penetration depth of 33.0 mm (±7.3 mm) and was similar to Dart 4 (Fig. 3). Dart 4 with no powder charge resulted in the shallowest mean (± SD) penetration depth of 27.0 mm (±5.6 mm) with 2.0 psi setting on the rifle. Dart 4 was selected for PIT delivery in mammals muscle tissue because it injected PIT the shallowest depth in the gelatin block while maintaining functionality. All PIT remotely delivered in prototype darts functioned properly in and after excision from the gel block.

### Remote delivery into tissue

Using Dart 4, we had a 90% success rate of PIT being injected from dart into muscle mass of cadavers at 2.5 psi setting for dart rifle although 2 PIT were implanted only in skin. Eighty percent of PIT actually injected in the muscle mass of cadavers with a mean (± SD) penetration of 22.2 mm (±3.8 mm) for those that could be accurately measured (*n* = 6); PIT were difficult to accurately locate for depth measurement in the hind quarters. Two PIT remained in the dart's needle upon impact but were successfully implanted on the second remote delivery attempt. We were able to read 95% of PIT in the cadavers after darting and frequency was able to be read after excision from muscle. One PIT could not be read in cadavers and was found to be broken upon recovery from muscle tissue.

### Longevity in a large mammal

We remotely delivered PIT into the hind quarter of 11 of 14 elk on the initial attempt. We successfully read PIT in the chute system of 13 of 14 elk within 48 hrs of successful injection; one elk was darted twice but PIT was never successfully read for unknown reasons. Of 13 elk successfully delivered PIT remotely, PIT was read monthly for up to 7 months on 9 elk. Of the remaining 4 elk, 3 had functioning PIT up to 5 months and currently reside at the captive facility. The PIT of the final elk was read at 3 months but was subsequently euthanized due to a positive test for chronic wasting disease. The euthanized elk was necropsied by a collaborator on QA 1487 that inadvertently failed to inspect injection site thus preventing recovery of the PIT. No PIT appeared to travel within the hind quarter but exact location could not be assessed in live animals. All elk in this study were maintained as a captive herd and will only be euthanized if positive for chronic wasting disease. In the future, any euthanized elk will have potential tissue damage by PIT examined during a diligent necropsy.

## Discussion

Delivery of PIT into large mammals was possible with prototype darts projected remotely using dart rifles currently used by veterinarians and wildlife biologists worldwide. Successful delivery of PIT was likely influenced by impact of the dart into the hind quarter. Proper psi setting was determined on cadaver muscle tissue but could be evaluated for other species to ensure proper impact of dart to successfully inject PIT. Alternatively, gel collars could be used on the dart needle to prevent premature dart ejection if immediate dart recovery is not necessary. Remote delivery of PIT is in its infancy stages of development and further considerations need to be explored prior to wide-scale use in large mammals.

### Requirements for marking methods in biological research

Gibbons and Andrews identified 6 points that should be considered before selecting a marking method for biological research [27]. Although subcutaneous injection of PIT has been used and evaluated on a variety of mammals [2], [10], [28], remote delivery of PIT and intramuscular injection has not been evaluated in relation to these 6 points. The 6 points to consider were: (1) no detrimental effects on study animal (i.e., behavior, survival, growth), (2) the mark should be permanent or last the duration of the study, (3) the code must be unique and easily readable, (4) all components of the marking technique should be durable and field hardy, (5) application and identification must be done to minimize handling time, and (6) marking equipment should not be cost prohibitive. The 6 points should be addressed in relation to remote delivery of PIT in tissue and could be the focus of future research.

#### No detrimental effects on study animal

A marking method “should not affect the behavior, physiology, growth, survivorship, or other biological traits of the individual, nor should it affect the behavior of other individuals with which the tagged individual interacts” [27]. The common practice of hand injecting PIT subcutaneously has no adverse effects on animals and we have no reason to believe any adverse effects of remote delivery of PIT into tissue would be any different. Remote darting with comparable-sized darts was conducted on multiple occasions throughout the year but showed no adverse effects on reproduction in free-ranging white-tailed deer used as controls in a contraceptive study [29]. Survival was not affected with remote injection of immobilizing drugs loaded into 3-cc double-barbed darts equipped with a 1.9 g transmitter and a battery unit [30]. Vaccines delivered in biobullets resulted in minimal injection-site trauma in white-tailed deer [20] or tissue damage was not detectable >30 days post-injection in beef cattle [21]. Furthermore, remote darting to deliver pharmaceuticals or immobilization drugs is a common practice, routinely approved by Institutional Animal Care and Use Committees and has not been shown to adversely affect individuals [25], [31]. Further research is needed on the influence of PIT on behavior of animals and potential for infection comparable to previous studies on remote darting in wildlife.

#### The mark should be permanent or last the duration of the study

Due to study duration, we were not able to evaluate whether the marks were permanent. We were able to determine, however, that proper PIT functioning persisted for up to 7 months in 75% of elk that were available for the duration of the study. Reasons for failure to read PIT in some elk were unknown and further research on PIT in tissue of live mammals is needed. Previous research has indicated that breaks in the polypropylene outer layer of PIT could allow body fluids to penetrate and damage electronics [32]. The remaining captive elk will continue to be monitored for several years but we have no reason to believe tissue will break down intact PIT and prevent proper function because the glass-encased tags are resistant to preservatives and animal decomposition [27]. Although only one PIT broke during the prototype dart and cadaver trials of our study, more durable PIT to prevent breakage of glass, combined with increased signal strength of PIT and readers would be a beneficial avenue of research to increase use of PIT in remote delivery to wildlife.

#### The code must be unique and easily readable

Similar to GPS and VHF technology, PIT codes are unique and a variety of instruments are available to read PIT. We successfully used the reader and a more water resistant and field durable reader (FS2001F-ISO) to detect individual PIT up to 50 mm in gelatin blocks and 26 mm in muscle tissue. Individual desert tortoises were monitored near culvert crossings under highways with an automated system that recorded unique frequency, date, and time of crossing up to 75 mm [1]. Individual mountain hare were implanted with PIT and were identified using feed troughs lined with a sensor array on all sides and a data logger up to 50 mm [2]. Adélie penguins were detected crossing weighbridges up to 1 m as they moved between breeding colonies in Antarctica [3]. Unique PIT frequencies can be read and identified with several types of PIT receivers at structures (i.e., flat plate readers, culvert rings), in unique habitats using an antennae array (multiplexing transceiver system; Biomark, Inc., Boise, ID, USA), or with handheld units at harvest check stations for game mammals. Multiple PIT reader systems are available to monitor uniquely identifiable (up to 1,000 frequencies per reader) fish, wildlife, structures, and habitats for a variety of research purposes [27], [33].

#### All components of the marking technique should be durable and field hardy

Only one of 20 PIT injected into the muscle tissue of cadavers broke upon impact and could not be read. We feel this may be related to dart manufacture causing a tight fit of the PIT in the dart needle. We had to bore out a few dart needles because PIT was not easily inserted into the dart. This is a simple problem to solve by the manufacturer or can be easily fixed in the field by testing for complete submersion of PIT into dart needle with no friction. Conversely, too loose a fit caused a few PIT to fall out of the dart during flight and were recovered on the ground within a meter of the target. Sealing the dart needle with an antiobiotic ointment solved this problem along with providing protection from potential infection. Further evaluation on remote delivery of PIT from greater distances from the target and higher psi settings would be needed to increase use of remote delivery unless an automated delivery system was designed [34]–[37].

#### Application and identification must be done to minimize handling time

The impetus for this study was the idea of marking large numbers of individuals in the field without the cost and hazards of capture typically required for marking large mammals with unique identifiers (i.e., GPS/VHF technology). No long-term methods currently exist for marking large mammals with unique identifiers that does not require capture (but see [11]). Marking free-ranging wildlife for survival analysis could be an added component of this method. Marker darts could be combined with PIT darts to individually identify animals with an ink dye to decrease repeated PIT injection during a marking season [38], [39]. Marking large numbers of mammals that are going to be harvested by the public (i.e., regulated state harvest) or by agencies as part of culling efforts could provide additional detail on location of kill and sex-specific survival dynamics. Capture and marking free-ranging wildlife is often cost prohibitive but remote delivery of PIT would enable increases in sample size, can occur at distances ≥10 m from the animal, and may be more aesthetically pleasing to the viewing public compared to radiocollars or ear-tags. Furthermore, automated devices have been designed for white-tailed deer to radiocollar with expandable collars, immobilize through remote darting, or treat with an acaricide to decrease Lyme disease that may be adapted for PIT for more effective delivery with less potential for trauma than remote darting using capture rifles [34]–[37].

#### Marking equipment should not be cost prohibitive

Darting rifles are quite common among researchers to capture large mammals and range from $500 to $2,000. The PIT readers vary in cost with the reader being the least expensive ($475) and a 6-antennae multiplexing transceiver system around $25,000 with each antennae capable of being placed out to 10 m from a central location. The PIT range in price depending on the quantity ordered was from $5 to $8 per PIT. The darts were prototypes at a cost of $6 per dart that would likely decrease if a market for PIT-delivery darts was established and mass production were initiated. Depending on study objectives, costs of marking with PIT seems relatively inexpensive, considering cost of capture for equipping with VHF/GPS collars, tracking equipment, personnel, and software often exceeds $50,000 to initiate a study of about 20 animals.

### Limitations of system

The impetus for the research was the perceived need of state-agencies to mark large numbers of free-ranging cervids for mark-recapture analysis that can be used to more accurately estimate population size. Although PIT has been used in a variety of research designs, delivery of PIT remotely also requires additional considerations to address practicality and animal welfare concerns. Further research is needed to determine PIT tag loss and retention, potential for post-harvest consumption of PIT, multiple injections per animal with PIT, potential tissue damage, and behavior of study animals beyond the time-scale of this study. Furthermore, readers of PIT would need to be explored to determine if study objectives could be achieved such as can study animals be funneled into an area to read injected PIT because of the limitations of PIT readers.

In conclusion, research on PIT has been occurring for decades but has not reached its potential for use in large mammals. Although several components of remote delivery of PIT still needs to be explored, remote delivery is possible, and proper function of PIT occurred for at least 7 months. If manufacturers of PIT could increase strength and durability of PIT for remote darting at greater distances and velocities, utility of PIT in wildlife research could increase. Furthermore, if manufacturers could increase the distance that readers can detect PIT beyond several meters, it could potentially expand the use of marking with PIT by researchers. Similar to remote darting for chemical immobilization and biobullet delivery, animal welfare is comparable while providing a valuable tool to mark large numbers of animals with minimal cost and hazards to biologists and study animals.
